# Identifying opportunities for timely diagnosis of bladder and renal cancer via abnormal blood tests: a longitudinal linked data study

**DOI:** 10.3399/BJGP.2021.0282

**Published:** 2021-12-14

**Authors:** Yin Zhou, Fiona M Walter, Luke Mounce, Gary A Abel, Hardeep Singh, Willie Hamilton, Grant D Stewart, Georgios Lyratzopoulos

**Affiliations:** Primary Care Unit, Department of Public Health and Primary Care, University of Cambridge, Cambridge, UK.; Primary Care Unit, Department of Public Health and Primary Care, University of Cambridge, Cambridge, UK; professor of primary care cancer research, Wolfson Institute of Population Health, Queen Mary University London, London, UK.; University of Exeter Medical School, Exeter, UK.; University of Exeter Medical School, Exeter, UK.; Center for Innovations in Quality, Effectiveness and Safety, Michael E DeBakey Veterans Affairs Medical Center, Houston, TX, US; Baylor College of Medicine, Houston, TX, US.; University of Exeter Medical School, Exeter, UK.; Department of Surgery, University of Cambridge, Addenbrooke’s Hospital, Cambridge, UK.; Epidemiology of Cancer Healthcare and Outcomes, Department of Behavioural Science and Health, Institute of Epidemiology and Health Care, University College London, London, UK.

**Keywords:** bladder cancer, diagnostic tests, routine, early detection of cancer, primary health care, renal cancer, test utilisation

## Abstract

**Background:**

Understanding pre-diagnostic test use could reveal diagnostic windows where more timely evaluation for cancer may be indicated.

**Aim:**

To examine pre-diagnostic patterns of results of abnormal blood tests in patients with bladder and renal cancer.

**Design and setting:**

A retrospective cohort study using primary care and cancer registry data on patients with bladder and renal cancer who were diagnosed between April 2012 and December 2015 in England.

**Method:**

The rates of patients with a first abnormal result in the year before cancer diagnosis, for ‘generic’ (full blood count components, inflammatory markers, and calcium) and ‘organ-specific’ blood tests (creatinine and liver function test components) that may lead to subsequent detection of incidental cancers, were examined. Poisson regression was used to detect the month during which the cohort’s rate of each abnormal test started to increase from baseline. The proportion of patients with a test found in the first half of the diagnostic window was examined, as these ‘early’ tests might represent opportunities where further evaluation could be initiated.

**Results:**

Data from 4533 patients with bladder and renal cancer were analysed. The monthly rate of patients with a first abnormal test increased towards the time of cancer diagnosis. Abnormalities of both generic (for example, high inflammatory markers) and organ-specific tests (for example, high creatinine) started to increase from 6–8 months pre-diagnosis, with 25%–40% of these patients having an abnormal test in the ‘early half’ of the diagnostic window.

**Conclusion:**

Population-level signals of bladder and renal cancer can be observed in abnormalities in commonly performed primary care blood tests up to 8 months before diagnosis, indicating the potential for earlier diagnosis in some patients.

## INTRODUCTION

Early diagnosis of cancer is associated with improved survival and patient-reported outcomes. However, timely detection of urinary tract cancers, in particular bladder and renal cancer, can be challenging in some patients.^1^ In the UK, clinical guidelines from the National Institute for Health and Care Excellence (NICE) exist to guide GPs on when to refer symptomatic patients with suspected cancer;^2^ however, these guidelines are often based on alarm symptoms. Early detection of cancer in patients without these symptoms can therefore be challenging.^1^^,^^3^ In patients with bladder cancer, longer diagnostic intervals were found, especially in females^3^^–^^7^ and those presenting without haematuria.^8^^–^^10^ Renal cancer is one of the cancers with rapidly rising incidence.^11^^–^^15^ With up to 60% of renal cell carcinoma presenting asymptomatically, and given it has been commonly associated with incidental diagnosis in recent decades,^16^ understanding the clinical scenarios triggering incidental identification would be useful.

Population-based studies have documented that, among some patients subsequently diagnosed with cancer, use of investigations starts to increase many months before the eventual diagnosis. This evidence highlights the presence of periods (or ‘diagnostic windows’) during which earlier diagnosis could, in principle, be possible for at least some patients.^17^^–^^20^ However, previous evidence has mostly focused on the use of tests (regardless of results), as opposed to whether tests were abnormal. Furthermore, evidence from well-characterised case series studies indicate that abnormal test results were commonly not followed up in patients subsequently found to have cancer.^21^^–^^23^ It is important, therefore, to study patterns of abnormal tests before the diagnosis; when prolonged intervals between abnormal tests and diagnosis occur, they may reflect missed diagnostic opportunities.

Motivated by these realisations, this study set out to find:

- The patterns of non-specific (‘generic’) abnormal blood tests commonly performed in primary care in the 12 months before diagnosis of bladder and renal cancer. Tests focused on included abnormal haemoglobin concentrations, high platelet count, raised inflammatory markers, and raised calcium that are known to convey a predictive value for cancer above what is expected by the patient’s age and sex in patients with non-specific symptoms.^24^^–^^28^ The aim of this aspect of the study was to document how often abnormalities in these commonly used blood tests could have triggered further investigations leading to shorter diagnostic intervals.
- The occurrence of abnormalities in ‘organ-specific’ blood tests such as raised creatinine and abnormal liver function tests that could have triggered investigation by subsequent imaging, potentially leading to incidental identification of renal cancer.

**Table:** How this fits in

Understanding which and when abnormal blood tests start to increase from a baseline rate in patients with bladder and renal cancer may highlight opportunities for more timely evaluation for cancer in some patients. This study found that commonly performed generic and organ-specific abnormal blood tests for bladder and renal cancer started to increase around 6–8 months before diagnosis. These findings suggest that there are population-level signals of bladder and renal cancer in commonly performed primary care blood tests, indicating potential for earlier diagnosis for some patients.

By examining patterns of abnormal blood tests commonly used in primary care, the study aimed to elucidate both the potential for earlier diagnosis in symptomatic patients and common clinical scenarios that may be leading to incidental identification.

## METHOD

### Data

Linked data from a primary care dataset, Clinical Practice Research Datalink (CPRD), and the National Cancer Registration Analysis Service (NCRAS) were used to examine the patterns of abnormal test results in a cohort of patients with bladder and renal cancer diagnosed between 2012 and 2015 in England. Details of data acquisition and cohort identification have been described in previous studies.^17^^,^^29^ Data on patients aged ≥25 years who were diagnosed with bladder and renal cancer between April 2012 and December 2015 were extracted from the CPRD. These data were linked at source to the cancer registry, from which additional patients were identified using International Classification of Diseases, Tenth Revision (ICD-10) cancer codes. The cancer registry diagnosis and date were used where available, and CPRD diagnosis and date in patients without linked data.

Cancers were subdivided into bladder, kidney, or upper urinary tract urothelial cell cancer (UTUC). Patients with UTUC were analysed separately as a result of possible difference in presentation from other patients with renal cancer, but the authors focused on the results of patients with bladder and renal cancer because of their larger sample sizes.

### Tests examined

The records of the patients included in the study were inspected for the use of primary care blood tests up to 12 months pre-diagnosis. Using the clinical experience of the authors and existing knowledge of associations between primary care blood markers and cancer,^14^^,^^16^^,^^27^^,^^28^^,^^30^ the pre-diagnostic patterns of the following abnormal tests (Box 1) were examined:

- Specific blood tests pointing to organ-specific abnormalities:
  — abnormal liver function test (LFT), including high aspartate aminotransferase (AST) and alanine transaminase (ALT); and
  — high creatinine.
- Generic blood tests:
  — full blood count (FBC) subcomponents: low and high haemoglobin, high platelet count, and high white cell count (WCC);
  — raised inflammatory markers including C-reactive protein (CRP) and erythrocyte sedimentation rate (ESR); and
  — raised calcium.

**Box 1.** Rationale for the blood tests examined

**Type of test**	**Test group**	**Specific test or component test examined**	**Rationale**
Generic	Full blood count	Haemoglobin (Hb)	Low Hb (anaemia) is a non-specific sign of renal and bladder cancer.^14^ High Hb (polycythaemia) may be associated with renal cancer as part of a paraneoplastic syndrome.^14^
		White cell count (WCC)	Raised WCC especially with lower urinary tract symptoms may be a clinical feature of bladder cancer.^27^
		Platelet	High platelet (thrombocytosis) may be a non-specific marker for cancer.^27^
	Inflammatory markers	C-reactive protein (CRP)	Raised CRP may be a non-specific marker for cancer.^27^
		Erythrocyte sedimentation rate (ESR)	Raised ESR may be a non-specific marker for cancer.^27^
	Others	Calcium	Raised calcium has been associated with increased risk of bladder cancer, as well as renal cancer by manifesting as a paraneoplastic syndrome.^14^

Organ-specific	Liver function test (LFTs)	Aspartate aminotransferase (AST) and alanine transaminase (ALT)	Abnormal LFTs can lead to subsequent imaging tests that reveal incidental renal cancers,^16^ and less commonly presenting as a paraneoplastic syndrome of renal cancer.^14^
	Renal function	Creatinine	Raised creatinine may be related to upper tract obstruction secondary to malignancy.

Local laboratory reference ranges, as captured in the CPRD test file, were used to define whether individual blood tests were normal or abnormal (low or high). Ambiguous results (because of incomplete reference ranges in the CPRD test file) were regarded as missing. The overall percentages of missing test results were low (0%–8%, with six of the nine tests having 0%–2% missing results), and therefore discarded (Supplementary Table S1).

### Analyses

For each test, the number of patients with an index abnormal test in each month up to 12 months pre-diagnosis was examined. The month immediately before diagnosis (that is, excluding month 1 pre-diagnosis) was excluded, because of the likelihood that these patients would have already entered the final stage of the diagnostic process for possible cancer.^17^^,^^25^^,^^28^ Each patient’s index abnormal test was defined as the first abnormal test within the 12-month period before diagnosis, concordant with prior literature.^17^^,^^29^

The inflection point at which the rate of abnormal tests increased above a baseline was then estimated. To do so, Poisson regression was employed, adjusting for age and sex, to model both the baseline rate of abnormal tests, and a departure from baseline occurring at the inflection point. Ten separate models were fitted, corresponding to 10 possible inflection points occurring at 2–11 months pre-diagnosis (months 1 and 12 were omitted owing to collinearity). All 10 models included data from all patients across the whole 12-month period with the outcome being the monthly count of abnormal results. Each model included a term to account for any baseline trend, and a second ‘inflection month’ variable to capture deviation from the baseline trend at different inflection points (one at a time, 10 in total, illustrated in Supplementary Box S1 and Supplementary Table S2). The inflection point associated with the best fitting model (that is, that with the largest log likelihood) was taken as the best point estimate for the month of departure from baseline. Bootstrapping was used to provide a confidence interval around this point.

Finally, a diagnostic window was estimated for each abnormal test during which potential further investigations could be initiated. This was calculated as the interval from the month at which the inflection point occurred, to the month immediately before diagnosis. The number of patients who had an early test, defined as a test that was performed in the first half of this diagnostic window, furthest back from diagnosis was examined. It was postulated that probable opportunities existed for a timelier diagnosis, especially during this early half of the diagnostic window. Where a diagnostic window included an odd number of months, the number of patients with an early test was calculated for the duration from the inflection point to the month immediately before the midpoint of the diagnostic window (for example, for an inflection point at 7 months pre-diagnosis, the first ‘half’ of the window was defined as 5–7 months inclusive; for an inflection point at 6 months, the first half of the window was defined as 5–6 months inclusive).

All analyses were performed using Stata (version 15).

## RESULTS

In total there were 5322 patients, consisting of 3398 (63.8%), 1715 (32.2%), and 209 (3.9%) patients with bladder cancer, renal cancer, and upper UTUC, respectively, who were initially identified from the dataset. There were 141 patients (2.6%) who had no tests recorded in CPRD and 648 patients (12.2%) had tests but not the studied tests in the 12 months before cancer diagnosis. Therefore, 4533 patients, consisting of 2890 (63.8%) with bladder cancer, 1465 (32.3%) with renal cancer, and 178 (3.9%) with upper UTUC who all had at least one of the 10 abnormal blood tests were further analysed. There were 3133 male patients and 1400 female patients who were aged between 25 and 101 years (median age 74 years, interquartile range 66–82 years) (data not shown).

The most common blood tests performed in the patients with bladder and renal cancer in the year before diagnosis were creatinine and FBC subcomponents (haemoglobin, WCC, and platelet count), in roughly 83% and 74% of patients, respectively. The highest proportion of abnormal results recorded were for raised inflammatory markers (CRP or ESR) (43%–45%), low haemoglobin (35%), and high creatinine (32%) (Supplementary Table S1). In general, there was no appreciable and consistent pattern of variation in abnormal tests by age and sex (Supplementary Figure S1 and Supplementary Table S3).

### Rate of abnormal tests by month

**Figure 1.**
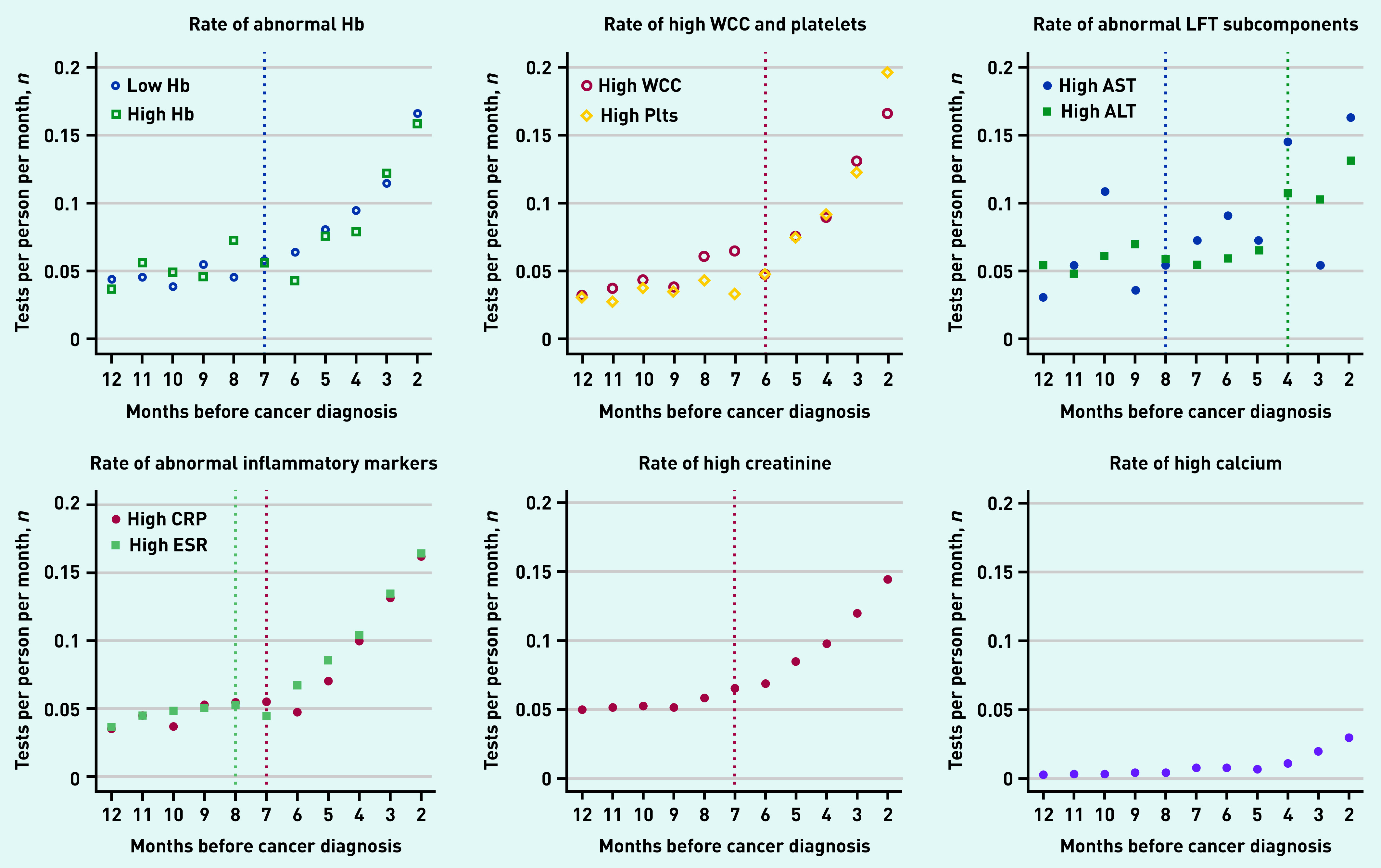
***Rate of abnormal blood tests in the year prediagnosis, with dotted line signifying increase in rate from baseline for that particular test (only statistically significant results shown).*** *^a^* *^a^* ***Inflection line for high WCC and high platelets both occur (and therefore overlap) at 6 months pre-diagnosis. ALT = alanine transaminase. AST = aspartate aminotransferase. CRP = C-reactive protein. ESR = erythrocyte sedimentation rate. Hb = haemoglobin. LFT = liver function test. Plt = platelets. WCC = white cell count.***

There was an increasing rate of abnormal tests for all tests towards diagnosis except high AST (likely because of the small number of tests performed) (Figure 1).

Evidence of inflection points for eight of the ten blood tests examined was found: low haemoglobin, high WCC, high platelet count, high CRP and ESR, high ALT and AST, and high creatinine (*P*<0.05; Figure 1 and Table 1). The earliest rate of increase was for high ESR and AST at 8 months prediagnosis, and the rate of increase was the closest to diagnosis for high ALT (4 months pre-diagnosis).

**Table 1.** Estimated inflection point for the increase in rate of abnormal blood tests and proportion of patients with an early test

**Test type (patients with that abnormal test, *n*)**	**Patients with a test during the diagnostic window, *n*^c^**	**Any cancer type^a^**		**Patients with an abnormal early test^b^**	
		**Month of increase (95% CI)**	***P*-value**	***n***	**%**
Low haemoglobin (*n* = 1253)	659	**7 (4.56 to 9.44)**	**<0.001**	**232**	**35.2**
High haemoglobin (*n* = 192)	123	5 (−0.27 to 10.27)	0.063^d^	—	—
High platelets (*n* = 391)	208	**6 (4.65 to 7.35)**	**<0.001**	**51**	**24.5**
High WCC (*n* = 573)	292	**6 (4.65 to 7.35)**	**<0.001**	**75**	**25.7**
High ALT (*n* = 302)	91	**4 (0.58 to 7.42)**	**0.022**	**30**	**33.0**
High AST (*n* = 39)	26	**8 (2.25 to 13.75)**	**0.006**	**11**	**42.3**
High CRP (*n* = 747)	426	**7 (4.72 to 9.28)**	**<0.001**	**120**	**28.2**
High ESR (*n* = 506)	321	**8 (4.54 to 11.46)**	**<0.001**	**82**	**25.5**
High creatinine (*n* = 1364)	686	**7 (3.92 to 10.08)**	**<0.001**	**289**	**42.1**
High calcium (*n* = 84)	39	5 (−0.38 to 10.38)	0.069^d^	—	—

a *Number of analysed patients is 4533 for all models. Bold text denotes results that are significant at* P<*0.05.*

b *Calculated for results where a statistically significant inflection point (*P<*0.05) is present.*

c *A diagnostic window is calculated from the inflection point to the month immediately before diagnosis. Where a diagnostic window included an odd number of months, the number of patients with an early abnormal test was calculated for the duration from the inflection point to the month immediately before the midpoint of the diagnostic window (for example, for an inflection point at 7 months pre-diagnosis, the first ‘half’ of the window was defined as 5–7 months inclusive; for an inflection point at 6 months, the first half of the window was defined as 5–6 months inclusive).*

d *The lack of evidence for an inflection point may arise because of an absence of an inflection point, a lack of power, or an inflection point occurring* >*11 months before diagnosis. ALT = alanine transaminase. AST = aspartate aminotransferase. CI = confidence interval. CRP = C-reactive protein. ESR = erythrocyte sedimentation rate. WCC = white cell count.*

### Proportion of early tests during diagnostic windows

Between one-quarter to two-fifths of all patients who had an abnormal result on ≥1 of the examined tests during the diagnostic window (from inflection point to the month immediately before diagnosis) had an early test, that is, one which was performed in the first half of the diagnostic window (Table 1). In particular, the highest proportion of patients who had an early test were patients with a high AST and raised creatinine (42% for each; Table 1), with this pattern also being consistent for individual cancer sites for raised creatinine (43% and 41% for bladder and renal cancer, respectively; data not shown). A lower proportion of patients with an abnormal generic test had the test early (for example, high platelets, high WCC, and high ESR — about 25% for each; Table 1).

## DISCUSSION

### Summary

Abnormalities in common primary care blood tests started to appear from 6–8 months before patients were diagnosed with bladder or renal cancer. Between 25% and 40% of patients with an abnormal test had the test performed in the early half of the diagnostic window, suggesting that opportunities might exist to initiate further investigations or referrals, and potentially expedite subsequent bladder or renal cancer diagnosis in at least some patients.

### Strengths and limitations

To the authors’ knowledge, this is the first study to examine the pre-diagnostic pattern of abnormal blood tests in patients with bladder and renal cancer, and when the abnormalities might appear before diagnosis. This study benefits from having a large sample size, with reliable coded information on test results. A major strength is that blood test results are automatically transferred to CPRD, therefore minimising any bias in recordings because of manual handling of the results. Finally, this method can also be used to examine patterns of pre-diagnostic tests and related abnormalities in other cancers.

This study assumes implicitly that inflection points in abnormal test findings occurred within a 12-month period. Although earlier inflection points are theoretically possible, this can be deemed unlikely from the observed findings. Furthermore, previous case–control studies looking at pre-diagnostic test patterns found that the majority of cancers were diagnosed in the year after the index test, and that cancer incidence returned to baseline in the second year after the index abnormal test.^25^

The sample size relating to patients with UTUC precludes precise estimations of associations. Results from patients with bladder and renal cancer were therefore focused on, in whom it is possible to make more reliable inferences because of their larger sample sizes. Although a statistically significant inflection point was found for abnormal AST, the confidence interval on this estimate is wide, reflecting the small sample size, and the exact estimate should be interpreted with caution.

This study did not examine presenting symptoms or other indications for the tests performed. Nevertheless, new abnormalities in either generic or organ-specific blood tests might represent situations where additional clinical explanations might be required, and further investigations or referrals are recommended.

### Comparison with existing literature

Building on prior evidence, the current study found that the monthly rate of abnormal primary care blood tests that are associated with increased risks for all cancers increased in patients with bladder and renal cancer in the months before diagnosis.^8^^,^^9^^,^^24^^,^^25^^,^^27^^,^^28^ In contrast to previous studies, the authors also examined organ-specific tests that could be associated with incidental detection of bladder or renal cancer, and identify when these abnormalities started to appear.

### Implications for research and practice

This study suggests that some patients with bladder or renal cancer could have their diagnosis expedited if abnormal tests led to definitive cancer investigation. Similar diagnostic windows (about 6–8 months pre-diagnosis) for both abnormal generic and organ-specific tests were found, suggesting that there may be opportunities to initiate earlier investigations for both types of abnormal tests depending on the clinical context. For the eight blood tests that demonstrated a rise in their baseline rates before diagnosis, at least one-quarter of the patients had the abnormality first detected in the early diagnostic window and prior to 3 months before diagnosis, a diagnostic interval threshold that could negatively affect survival in some patients with bladder and renal cancer.^31^^,^^32^

These findings suggest that there may be greater propensity to improve evaluation of abnormal organ-specific tests than generic tests. The study found that 33%–42% of patients with abnormal LFTs and creatinine have an abnormality early in the diagnostic window, a relatively high proportion of patients with an early abnormal test, suggesting that opportunities for more rigorous evaluation of abnormal organ-specific tests might exist in some patients, especially in the presence of other risk factors such as age and smoking status. Symptomatic presentation of renal cancer has been associated with advanced disease, whereas incidental diagnosis has been reported during investigations for pre-existing or non-urological cancer clinical features, including for hepatobiliary causes or urinary tract obstruction.^16^^,^^33^ It is possible that, with improved direct access in primary care to imaging such as ultrasound, further investigations of abnormal LFTs and creatinine can result in a continued increase in incidental, and therefore early-stage, diagnosis of renal cancer.^34^

When considering the generic tests, earlier investigations could nevertheless be triggered in at least some patients, given that about one-quarter of patients with an abnormal generic blood test also had the abnormality first detected in the early half of the diagnostic window. It is possible that the generic tests representing markers of inflammation might be associated with more symptomatic disease, and are more likely to result in further active monitoring or investigations that subsequently led to a cancer diagnosis.

Finally, it is important to note that, although this study found diagnostic windows during which further investigations for abnormal results could potentially be initiated, it does not illuminate whether this clinical behaviour should happen. Many of the abnormal tests have low positive predictive values (PPVs) for cancer and would not qualify for urgent investigation under current UK clinical guidance, which currently suggests investigations for clinical features with PPVs above 3%.^2^ They should be considered in combination with other patient and clinical risk factors and clues, including the age of the patient, duration, severity, and presence of other symptoms and signs. Although it was not possible to examine the indications leading to the performance of each abnormal test, the findings of this study suggest that changes in the level of abnormal tests from a background rate exist in patients subsequently diagnosed with bladder and renal cancer, either in response to symptomatic disease or through routine blood monitoring carried out for other reasons. Should the current NICE guidance be liberalised and the referral threshold reduced, this study suggests there is considerable potential for earlier diagnosis of bladder and renal cancer. Further studies that examine the PPV of the examined tests for bladder and kidney cancer are also necessary to guide clinicians on the most appropriate subsequent management plans following abnormalities in these tests.

In conclusion, the findings demonstrate that abnormalities in commonly performed primary care blood tests represent population-level signals of bladder and renal cancer that can be observed up to 8 months before diagnosis, suggesting that there may be opportunities to expedite the diagnosis in some patients. There is a need to further evaluate associations between abnormal tests and bladder and renal cancers for individual patients, and to consider the clinical context in which these tests are performed, to better understand the clinical utility of these common tests in the early identification of symptomatic cancer.

## Data

Data available from authors on request.
